# Supplementary material for: Are carbon emissions trading and green financial instruments synergistic? -Comprehensive quantitative research based on content analysis
Source: PLoS One. 2024 Mar 7;19(3):e0298601. doi: 10.1371/journal.pone.0298601 (PMC10919601; doi:10.1371/journal.pone.0298601)
Supplement: S1 Data — (ZIP) [file pone.0298601.s001.zip › Data/地级市Z_F工作报告.zip!/║■─╧/╙└╓▌╩╨/└┤╫╘╣1⁄2╓┌║┼í╛┬φ┐╦╩2╛▌═°í┐_2016.docx]

各位代表：

　　现在，我代表市人民政府，向大会报告政府工作，请连同《永州市国民经济和社会发展第十三个五年规划纲要（草案）》一并审查，并请各位政协委员和其他列席人员提出意见。

　　一、“十二五”工作回顾

　　“十二五”时期，是我市经济社会发展极为重要、很不平凡、富有成效的五年。面对错综复杂的宏观环境和艰巨繁重的改革发展稳定任务，全市人民在市委的坚强领导下，顽强拼搏、锐意进取，总体完成了“十二五”规划确定的目标任务，开启了中国梦的永州新篇章。

　　经济实力大幅提升。地区生产总值2012年跨上千亿台阶，2015年达到1413亿元，五年年均增长10.5%。规模工业增加值达到330亿元，年均增长13.8%。财政总收入达到131.1亿元，年均增长19.6%。固定资产投资达到1535亿元，年均增长28.4%。社会消费品零售总额达到527亿元，年均增长14.4%。金融机构各项存款余额达到1573.1亿元、贷款余额达到793.62亿元，分别是2010年的2.17和2.19倍。全面小康实现程度达到84.6%，五年提高15.6个百分点。三次产业结构由24.8：36.3：38.9调整为21.3：37.0：41.7。现代农业初现雏形。粮食、烤烟、蔬菜、水果、畜禽水产等大宗农产品生产增量提质，农业产业化保持全国先进，国家、省级龙头企业由21家增至36家，以永州国家农业科技园为代表的现代农业园区发展迅速。成功跻身全国油茶产业发展示范市。新型工业化稳步推进。规模工业企业达到794家，其中年产值过亿元企业320家、过10亿元企业6家，分别比2010年增加208家和3家。规模以上农产品加工业产值突破200亿元，装备制造、矿产品加工、轻纺制鞋、电子信息产业建成百亿产业，新能源新材料、生物医药等新兴产业来势较好。省级工业园和工业集中区由7个增加到11个，永州经开区、祁阳经开区建成百亿园区。第三产业快速发展。文化旅游产业加速融合，“古城名山”建设稳步推进，新增3A级景区11家、4A级景区2家，宁远、双牌跻身全省旅游强县。金融业、房地产业等发展迅速，电子商务、现代物流等新兴服务业加速起步。质量强市成效明显。新增国际商标3件、中国驰名商标4件、国家地理标志保护产品2个、“国字号”质量检测中心1个，荣获省长质量奖1个、省质量管理奖5个。县域经济实力增强。地区生产总值过百亿县区由3个增加到7个，其中祁阳、冷水滩、零陵跨过200亿元台阶；财政总收入过10亿元县区由“零记录”发展到5个，所有县区财政收入实现五年翻番。祁阳、宁远、双牌、江华被列为全省特色县域经济重点县，冷水滩、蓝山、道县被评为全省县域经济发展先进县，江华、新田被评为全省全面小康建设提升幅度十快县，零陵被评为全省全面小康经济快进县。非公经济活力释放。占GDP比重达到58.3%，支撑发展、吸纳就业、增加财税、改善民生的作用不断增强。

　　城乡面貌显著改观。交通进入高铁时代。湘桂高铁2013年开通运行，目前已实现动车直达北京、上海、广州、深圳，永州融入长株潭一个半小时经济圈、珠三角三小时经济圈；厦蓉、道贺高速永州段和二广高速永州至广州段全面建成通车，全市高速公路通车里程达到489公里，居全省第5位，11个县区全部实现半小时内上高速；祁冷一级公路及一批干线公路、出省跨境公路建成通车；零陵机场开通深圳、昆明、上海航线，综合立体交通网络基本形成。城镇彰显崭新风貌。中心城区扩容提质强力推进，建成区面积由54.8平方公里拓展到60.1平方公里，人口由54万增加到63万，成功创建国家卫生城市、国家森林城市。县城和小城镇面貌变化显著，集聚辐射能力增强，人口过10万的县城达到6个，其中过15万的县城达到3个、过20万的县城达到2个，新增人口过5万的小城镇3个，江华荣获全国文明县城称号，宁远成功创建国家卫生县城，黄田铺、回龙圩等18个乡镇进入国家、省级重点乡镇示范，全市城镇化率由35.38%提高到45%。农村焕发生机活力。新农村建设扎实推进，创建全国美丽乡村试点村1个、省级新农村和美丽乡村示范村23个，全市配备农村保洁员9000余人，86%以上的村实现清扫保洁，农村生活垃圾集中处理率达到80%，卫生厕所普及率达到71.82%，祁阳、宁远、蓝山被评为全省新农村建设示范县。农村交通条件大幅改善，完成公路畅通工程4142.5公里，新增通水泥路行政村855个，除11个村因水库建设影响暂缓实施畅通工程外，其余所有行政村实现通水泥（沥青）路。农田水利建设力度空前，五年完成水利投入103亿元，是“十一五”的3.05倍，涔天河水库扩建及灌区工程相继开工建设，病险水库除险加固、中小河流治理、小农水重点县、水土保持和“五小水利”项目建设取得显著成效。新建高标准农田36.26万亩。全市实现国家农业综合开发全覆盖。能源信息建设全面加强。神华国华永州火电开工建设，祁阳浯溪、湘祁、双牌五里牌等水电站建成投产。风电开发形成热潮，已批装机和在建规模全省第一。城乡电网不断完善。“气化永州”稳步推进。信息网络得到加强。祁阳被列为全国智慧城市试点县。

　　改革开放深入推进。改革走出永州路径。行政管理体制改革走在全省前列，在全省率先推行“三清单一目录”管理，简政放权得到落实。商事制度改革走在全省前列，在全省率先推行“先照后证”、“三证合一”、“一照一码”，创新创业活力极大迸发。农村产权制度改革走在全省前列，土地承包经营权确权登记全面完成，流转平台规范运行，抵押贷款稳步推进。农村信用社改革走在全省前列，全市9县农信社全部改制为农村商业银行，中心城区组建永州农商行进展顺利，交通银行、华融湘江银行、长沙银行、省农信担保公司相继进驻。投融资体制改革取得突破，组建了城投集团、经投集团等投融资公司，推行了PPP项目试点，融资能力和水平显著提升。社会治理机制改革深入推进，网格化建设“永州模式”在全省推广。市属国企改革、市县区政府机构改革、国有林场、集体林权制度改革全面完成，并乡合村、财税体制、不动产统一登记制度等改革稳步推进。开放取得重大成效。湘南承接产业转移示范区于2011年获批设立，永州发展纳入国家战略层面。五年累计到位外资31.8亿美元，引进内资1005.2亿元，承接产业转移项目2237个，完成外贸进出口总额27亿美元。新增3个省级出口基地。海关、商检永州办事处相继设立，公路口岸、铁路口岸获批建设。

　　人民生活大为改善。城乡居民人均可支配收入分别达到21889元、10810元，年均分别增长10.6%和13.6%。连续五年全力为民办实事，一批事关群众切身利益的问题得到较好解决。就业水平不断提高，五年新增城镇就业29.2万人、农村劳动力转移就业30.56万人，帮助下岗失业人员再就业3.95万人次。社会保障体系不断完善，“五项保险”参保人数达到629.6万人次，城乡低保基本实现应保尽保，五保供养水平逐年提高，在全省率先开展新农合即时结报和城乡居民大病保险。住房保障得到加强，累计开工建设保障性安居工程和各类棚户区改造15.3万套，建成10.8万套。改造农村危房8.75万户。扶贫工作成效明显，五年累计扶助贫困人口脱贫53万人。新解决农村人口饮水安全问题184万人。新建、改造农贸市场117家。人民群众衣食住行提档升级，家庭汽车拥有量从6.8万辆增加到17.1万辆。生态建设强力推进，水、空气质量居全省前列，湘江源头区域获批国家生态文明先行示范区，新田、宁远、双牌、蓝山、江华列入国家主体功能区建设试点示范，九嶷山、舜皇山创建国家级自然保护区，福音山获批国家森林公园，新增5个国家湿地公园、3个国家水利风景区。五年完成人工造林259.2万亩，森林蓄积量净增1079万立方米，森林覆盖率提高4.71个百分点。江华被评为全国绿化模范县，金洞被列为国家储备林示范基地。大力淘汰落后产能，全力加强垃圾、污水处理等环保基础设施建设，城市生活污水处理率达到92.3%，生活垃圾无害化处理率达到97.7%。

　　社会事业全面进步。教育优先得到确立。新增城区义务教育学位8.51万个，其中中心城区3.2万个，新增农村公办幼儿园55所，新建义务教育合格学校564所，改造中小学危房346万平方米，新增教师周转房5225套。名校名师名校长工程建设取得实效，教育教学质量大幅提升。科技创新成果丰硕。组织实施省级以上科技项目257项，专利授权4047件，新认定国家高新技术企业68家，创建国家级企业工程技术研究中心1个、国家创新型试点企业2家，成功跻身国家知识产权试点城市，祁阳、冷水滩、东安被评为全国科技进步先进县。医疗卫生事业快速发展。医疗卫生体制改革稳步推进，医疗保健基础设施显著改善，重大疾病防控和卫生应急能力不断提升。宁远、江华创建成全国中医药工作先进县。计划生育工作五年保持全省先进。文化强市建设扎实推进。新增全国重点文物保护单位18处、总量达到26处，排全省第二位。大批传统村落、历史文化街区、非物质文化遗产列入国家、省里名录。成功举办两次全省公祭舜帝大典和一次世界舜裔宗亲暨永州市社会各界公祭舜帝大典活动。文化体育基础设施进步明显，群众性文化体育活动和精神文明创建活动蓬勃开展，竞技体育实现奥运金牌零突破。国防教育、国防动员、人民防空工作取得新成绩，驻永部队和民兵预备役部队为地方发展作出新贡献。工青妇等群团组织作用充分发挥。民族宗教、外事侨务、新闻出版、审计、统计、史志、科协、气象、地震、档案、对台、老龄、关工委、残联、慈善等工作全面进步。

　　民主法制不断加强。认真执行市人大及其常委会的决议、决定，自觉接受人大及其常委会的法律监督、工作监督，主动接受市政协的民主监督，广泛听取各民主党派、工商联、无党派人士和人民团体的意见、建议。五年办理人大代表议案、建议951件，政协委员提案1046件，办理结果满意率逐年提升，创国卫、创国森、闲置土地清理、小区整治、湘纸搬迁等一批重点议案、建议和提案的办理，效果良好。大力推进简政放权，市政府工作部门保留行政职权2527项、市本级行政许可事项缩减至124项，均为全省最少的市州之一，审批时限在法定时间内缩短“两个1/3”以上。完善政务服务体系，积极推进网上政务服务和电子监察系统应用。切实加强基层民主，全面落实依法行政。积极开展普法宣传，“六五”普法圆满完成。加强惩治和预防腐败体系建设，强化行政监察和审计监督。扎实开展创先争优活动、党的群众路线教育实践活动和“三严三实”专题教育，推出两个全国重大先进典型和一个全省重大先进典型。坚决反对“四风”，不断改进作风。积极推进政府绩效考核，强化领导干部实绩管理与执行力考核，政府执行力和公信力不断增强。

　　刚刚过去的2015年，是“十二五”收官之年，我们认真落实市委的决策部署，主动适应新常态，持续推进“四个三”，大力实施“八大计划”，奋力破解“五大难题”，全力走好“永州路径”，扎实办好“六件惠民实事”，较好完成了市四届人大六次会议确定的目标任务，为“十二五”划上了圆满的句号。

　　着力稳增长，发展势头稳中向好。主要经济指标增速高于全省平均水平，在全省排位位次前移，多数居于第一方阵。预计，地区生产总值增长9%，排全省第6位，同比前移3位；规模工业增加值增长10.2%，增速自2014年6月份以来18个月排全省第1位；固定资产投资增长17.8%，排全省第11位；社会消费品零售总额增长12.1%，排全省第7位；财政总收入增长15.6%，排全省第3位；城乡居民收入分别增长8.5%和9.5%，分别排全省第8位和第10位；全面小康实现程度提升4个百分点，排全省第2位。农业发展势头良好，实现粮食总产318.16万吨、水果108.8万吨、蔬菜450万吨，收购烟叶55.6万担，出栏生猪970万头，新建农业标准化示范基地90个、粮食万亩高产示范片51个，新增农产品加工龙头企业26家、“三品一标”产品75个、省著名商标10个，东安被评为全国农产品质量安全县，道县、江永、新田获批国家出口食品农产品质量安全示范区。着力实施规模工业企业转型升级计划和全民创新创业计划，规模工业总产值突破千亿大关，新发展小微企业1600家，新入统规模企业127家、排全省第一。零陵烟厂技改完成，产值达到67亿元。长丰猎豹汽车新车型CS10量产上市，全年整车产量达到历史最高的3.8万台，销售收入达到40亿元。重点产业和新兴产业发展提速，生物医药、先进装备制造、电子信息产业产值分别增长25%、38.6%、44.2%。坚持科技创新驱动，组织实施省级以上科技计划项目51项，授权专利931件，新增国家高新技术企业13家。大力实施百亿园区推进计划和创新创业园区“135”工程，全市园区新建标准厂房220万平方米，引进“双创”企业150家，均居全省前列，新增省级新型工业化示范园区1个、省级创新创业示范基地4个。文化旅游产业持续升温，“零陵古城”保护项目开工建设，阳明山挂牌国家生态旅游示范区和海峡两岸交流基地，九嶷山创建国家级风景名胜区通过国家住建部评估，江华瑶族自治县成功举办60周年县庆暨中国第十三届瑶族盘王节，东安创建首个“中国德文化之乡”。房地产业健康发展，新建城镇住房462.78万平方米，完成房屋交易金额76.87亿元。电子商务发展迅速，淘宝“特色中国·永州馆”上线运行，在全国首个实现各县区同步推进阿里巴巴农村淘宝项目，江永跻身国家电子商务进农村综合示范县。开放承接势头良好，全年承接产业转移项目542个，实际利用外资8亿美元，到位内资196亿元，实现进出口总额8.5亿美元。长沙海关永州办事处正式挂牌，湖南农副产品集中验放场全面竣工。

　　着力抓项目，发展动能加速集聚。重大基础设施项目进展较好，207国道双牌至道县段改建工程竣工通车，冷水滩至东安、零陵至双牌一级公路开工建设，新建农村公路605公里、农村招呼站155个，永州至深圳直达高铁、至上海民航班机开通；涔天河水库扩建工程超进度推进、灌区工程正式开工、库区移民统一迁建签约率达97.5%，毛俊水库项目建议书获国家水利部批复；神华国华永州火电一期工程快速推进，江华大路铺、东田、江永黄甲岭等风电场并网发电，蓝山四海坪、道县洪塘营、零陵石岩头等风电项目开工建设，新田220千伏智能化变电站和冷水滩岚角山等3个110千伏变电站竣工投运，祁阳梅溪110千伏变电站开工建设；智慧城市创建计划高位破题，市云计算中心投入试运行，数据库建设加紧推进，综合应用平台部分子系统上线运行，4G等通讯基础设施建设进展较好。产业项目建设成效显著，恒伟医药、康都制药、德福隆电子、九恒集团、千山药机等项目竣工投产，五矿江华稀土产业园、市烟草产业园、科力尔电机、温氏集团在永生态养殖等项目进展顺利。城建项目建设快速推进，中心城区实施重点项目148个，完成投资80亿元，15条主次干道、40条背街小巷提质改造和梅湾路、人民路等建筑立面整体改造完工，23条断头路全部通车，19个安置小区建设实现质的突破。国家历史文化名城申报和交通管理模范城市创建工作扎实推进，道县、江永、蓝山成功创建省级卫生县城。项目开发工作卓有成效，全市共开发包装“十三五”重大项目790个，总投资超过1万亿元，其中235个单列项目初步纳入省“十三五”规划项目库，占全省项目总数的10%以上，在全省14个市州中排名第一。

　　着力解难题，发展瓶颈有效缓解。坚持以改革增活力，行政管理体制改革迈出大步，出台2015年版“三清单一目录”，在全省发出首张全国统一编码的“三证合一、一照一码”营业执照，全年新增各类市场主体3.1万户，其中新设立私营企业4486户；农村产权制度改革纵深推进，县、乡两级土地流转服务中心全面组建到位，新增新型农业经营主体2569个、农地流转面积80万亩；社会治理机制改革落地落实，社区网格化全面推进，改造、新建63个社区服务用房，服务群众能力进一步提升。加大跑项争资力度，全年争取中央、省补助资金244.1亿元，新增、置换债券额度51.99亿元，盘活财政存量资金41.48亿元；引导金融机构扩大信贷投放，全年新增银行贷款152亿元、同比多增65.14亿元，存贷比达到50.45%；发挥地方投融资平台作用，市城建投、经建投、开建投分别融资68亿元、38亿元、14亿元，创历史新高；加强政府与社会资本合作，中心城区供水和污水处理、生活垃圾焚烧发电等PPP试点示范项目相继启动；加强对融资担保、小额贷款等投资类公司的有效监管，稳妥处置了民间融资引发的群访事件，有效防范了区域性经济风险。加大用地保障力度，全市获批建设用地8.41万亩，排全省第2位；成功处置闲置土地1.48万亩，占应处置面积的98.2%；中心城区征收土地3901亩，拆迁房屋49.2万平方米，拆除违法建筑40.45万平方米。通过上述工作，较好破解了一些瓶颈制约，有力保障了经济社会发展的要素需求。

　　着力增福祉，发展成果惠及民众。加大民生投入，全市民生支出占财政总支出比重达到78.8%。扎实办好民生实事，省定24件为民办实事项目和市定6件惠民实事全面完成。坚持就业优先，全年新增城镇就业5.87万人、农村劳动力转移就业6.84万人，发放小额担保贷款3.7亿元，扶持6300多人成功创业。提高社会保障水平，城市低保月人均救助水平达到246.6元，农村低保月人均救助水平达到109.3元，五保对象年分散供养标准达到2908.8元，均达到或高于省定标准。发放“五项保险”待遇60.55亿元，社会保障卡综合应用纳入全国试点示范。建设保障性住房和实施各类棚户区改造4.75万套，改造农村危房2.03万户。改造农贸市场28家。全面部署精准扶贫攻坚战，931个贫困村实现驻村帮扶工作队全覆盖，全年减少贫困人口12万、贫困村59个。坚持生态优先，新增人工造林40.11万亩，湘江纸业停产关闭，湘江保护和治理第一个“三年行动计划”基本完成，湘江干流出境水质保持二类标准，各项环保约束性指标全面完成。坚持教育优先，新增城区义务教育学位2.1万个，其中中心城区0.61万个，新建义务教育合格学校56所、农村公办幼儿园23所、教师周转宿舍1800套。大力发展医疗卫生事业，全面推进县级公立医院综合改革，涉改9县全部实施药品零差率销售。食药监管得到加强。深入推进平安永州建设，扎实抓好安全生产、信访维稳、社会治安综合治理，组织开展严打整治百日会战、严打盗骗毒等系列专项行动，社会治安持续向好，重大案件全部侦破，打黑除恶、网上追逃、刑事案件破案率排名全省第一，安全生产、综治工作考评分别跃居全省第2位、第3位。

　　各位代表，过去的五年，我们应对重大挑战，经受重大考验，取得重大成就，实现重大跨越。五年来，特别是近三年来，市委团结带领全市人民励精图治、奋发图强，攻克了一个又一个难关，夺取了一个又一个胜利，办成了一批大事，解决了一批难事，把一些偶然变成了必然，把一些不可能变成了可能，全市上下呈现出“人心思进、发展提质、事业兴旺、社会和谐”的良好态势，永州站在了一个新的历史起点上，正以新的面貌、新的姿态、新的形象阔步迈向第一个百年奋斗目标。

　　这些成绩的取得，是上级党委、政府亲切关怀、高度重视的结果，是市委正确领导、科学决策的结果，是市人大、市政协加强监督、大力支持的结果，是全市人民加压奋进、团结拼搏的结果。在此，我谨代表市人民政府，向全市人民，向驻永部队、武警官兵，向中央、省驻永单位，向所有关心支持永州建设发展的各级领导、各界朋友，表示衷心的感谢！

　　五年取得的成绩来之不易，积累的经验弥足珍贵。我们深深地感受到，永州的发展之所以有今天的大好局面，主要在于：这些年来，我们始终坚持充满激情干工作，勇往直前攻难关，有效引领了全市人民的发展信心；始终坚持凝心聚力干大事，齐心协力解难题，较好突破了方方面面的发展瓶颈；始终坚持胸怀永州谋全局，立足当前利长远，科学确立了切合实际的发展思路；始终坚持咬定目标不放松，一张蓝图绘到底，成功获得了实实在在的发展成效；始终坚持实绩面前论英雄，主战场上辨优劣，真正形成了创先争优的发展氛围；始终坚持贴心为民谋福祉，百姓之事大于天，全面贯彻了以人为本的发展要求。对此，我们务必倍加珍惜、矢志坚持、大力弘扬。

　　在肯定成绩的同时，我们也清醒认识到，政府工作与全市人民的期待还有差距，经济社会发展还面临不少困难和问题。主要是：经济整体实力不强，发展不快、总量不大、均量不高、质量不优问题仍较突出，企业生产经营状况整体不容乐观，经济下行压力仍然较大，全面建成小康社会任务艰巨；发展基础不牢、后劲不足，新型工业化薄弱、基础设施薄弱、中心城市带动力薄弱问题没有得到根本改变；社会事业短板较多，改善民生还做得不够，扶贫攻坚任务十分繁重，部分群众生产生活还比较困难，影响社会和谐稳定的因素仍然较多；政府职能转变、干部工作作风与新常态、新形势的要求还不相适应，一些地方和部门消极腐败、为官不为现象仍然存在。对这些问题，我们将高度重视，积极研究和采取有效措施，切实加以解决。

　　二、“十三五”总体部署

　　“十三五”时期是我市全面建成小康社会的决胜期，是实现后发赶超、转型跨越的关键期。放眼全球，世界经济在深度调整中曲折复苏，新一轮科技革命和产业变革蓄势待发；综观全国，我国经济发展进入新常态，长期向好的基本面没有改变，仍处于大有作为的重要战略机遇期；环顾全省，依托“一带一部”区位优势和多个国家层面的战略支持，当前发展风生水起，未来发展空间广阔；审视自身，经过多年打基础增后劲，我市经济硬实力、发展软实力、综合竞争力迈上新台阶，支撑经济社会发展的利好因素不断集结。

　　面对新形势，抢抓新机遇，全市“十三五”发展的指导思想是：高举中国特色社会主义伟大旗帜，全面贯彻党的十八大和十八届三中、四中、五中全会精神，以马克思列宁主义、毛泽东思想、邓小平理论、“三个代表”重要思想和科学发展观为指导，深入贯彻习近平总书记系列重要讲话精神，落实“五位一体”总体布局和“四个全面”战略布局，坚定贯穿“五大发展”理念，坚持发展第一要务，坚持以提高经济发展质量和效益为中心，着力稳增长、促改革、调结构、打基础、惠民生，深入实施“四个三”工作部署，推进“五化”同步，促进“三量齐升”，加快形成引领经济发展新常态的体制机制和发展方式，加快建设生态引领、创新涌流、产业发达、生活殷实、城乡靓丽的品质活力永州，确保如期全面建成小康社会。

　　战略定位是：建设生态文明城市、全国文明城市、区域性铁路枢纽城市、湖南对接东盟桥头堡、湘南承接产业转移新高地，着力打造品质活力永州。

　　主要目标是：经济实力更趋雄厚，社会发展更具品质，民主法治更臻完善，生态环境更为优良，人民生活更加殷实，努力实现“两快于一翻番”：即经济增速快于全省平均水平，城乡居民收入增速快于经济增速，财政总收入力争实现翻番。

　　落实上述指导思想，实现新的目标定位，核心是要咬住“四个三”工作部署不动摇，保持定力、深度用力、精准发力，在深度推进和持续实施中，把握年度内容和特点；关键是要紧密结合永州实际，创造性地贯彻落实创新、协调、绿色、开放、共享“五大发展”新理念，切实以发展新理念，引领发展新实践，开辟发展新境界。

　　实现经济实力新跨越。保持经济平稳较快增长，年均GDP增速达到9%左右，到2020年，全市地区生产总值达到2500亿元左右，人均GDP超过43000元，固定资产投资、社会消费品零售总额、规模工业增加值分别年均增长16%、12%和10%。三次产业结构调整为17：39：44，高新技术产业增加值占地区生产总值比重上升5个百分点。现代工业更加壮大，规模工业总产值突破2000亿元、增加值突破550亿元，实现农产品精深加工产业产值过500亿元，先进装备制造、电子信息产业产值过300亿元，矿产品精深加工、轻纺制鞋产业产值过200亿元，生物医药、新能源新材料、建材建筑、现代物流产业产值过100亿元，新增规模工业企业500家、总数达到1000家，培育年产值过亿元企业200家、过10亿元企业15家、过50亿元企业4家、过100亿元企业3家，全市12个工业园和永州国家农科园全部建成百亿园区，永州经开区打造国家级经开区。现代农业更具优势，确保全市耕地保有量保持在500万亩以上，打造粮油、畜禽水产、果蔬、油茶、毛竹、家具六大百亿产业，创建一批名牌企业和产品，主要农产品加工转化率达到60%，形成年销售收入过10亿元的龙头企业5家、过亿元企业100家，60%以上的农户进入农业产业化经营领域，初步构建粮经饲统筹、农林牧渔结合、种养加一体、一二三产业融合的现代农业产业体系。现代服务业更显活力，实施服务业倍增计划，推进服务业创新发展，突出抓好文化旅游、现代物流、金融保险、房地产业，促进互联网与服务业融合，培育新兴服务模式和服务业态，打造国内知名人文山水旅游目的地、湘粤桂边界核心商贸流通枢纽、知名健康养老和休闲养生之城。

　　推动基础设施新突破。提升立体交通网络。统筹抓好铁路、公路、水运、机场、城市公共交通建设，积极推进衡永高速公路、洛湛铁路邵永客运专线、湘桂铁路既有线电气化改造、兴永郴赣及桂永郴赣铁路新建、永州北铁路货站、永州火车站提质改造、零陵机场迁建、湘江永州段千吨级航道改造及港口、湘桂运河等重大项目，大力推进市域快速通道、农村公路、旅游快速通道等建设，构建更加方便快捷的立体交通网络。强化农田水利建设。全面建成涔天河水库扩建、毛俊水库等一批重大水利工程，力争开工建设何仙观等水库，抓好涔天河、双牌、江永古宅水库等大中型灌区建设改造，全面完成中小型病险水库、大中型病险水闸除险加固和城市防洪工程建设，继续加强“五小水利”、中小河流治理、小农水项目、水土保持和山洪地质灾害防治。大力实施城市、农村饮水安全工程。推进高标准农田示范工程建设，建成集中连片、旱涝保收高标准农田160万亩。健全能源供应体系。实现神华国华永州火电项目一期工程竣工投产、二期工程动工兴建。推进能源互联网和智能电网建设，继续抓好农村电网改造升级。加快风电资源开发，力争新建、投产风电装机规模180万千瓦。合理布局光伏电站。实施小水电增效扩容改造工程。推进“气化永州”战略。完善信息基础设施。大力推进“三网融合”，打造光网无线城市，建设“宽带乡村”，建成北斗位置导航（湖南）数据中心、云计算中心等信息应用平台，加快构建“智慧永州”。

　　建设两型生态新家园。着力打造新型城镇。实施“一核一群一带一圈”战略，优化区域城镇发展格局。着力做强“一核”，加快推进零、冷两区一体化，加快构筑“一主三副”城市构架，实现组团发展，尽快做大做强中心城市，真正昂起市域城市“龙头”；加快推进“一群”，以基础设施互联互通、产业建设分工协作为重点，加快祁阳、东安、双牌与中心城市的融城步伐，全力打造富有生机、充满活力的潇湘城市群；精心搞活“一带”，以二广高速公路为轴线，以沿线城镇、园区为依托，积极承接产业转移，突出宁远、新田、蓝山发展，建设二广经济增长带；稳步培育“一圈”，以道县、江华、江永为支点，发挥农业、生态、旅游和民族文化优势，在市域西南部培育形成生态经济圈。支持祁阳、道县撤县设市。加强以中心镇为重点的小城镇规划建设。建立城乡统一的户口登记制度，全面放开中心城区、县城、管理区和乡镇政府所在地的落户限制，实行财政转移支付和城镇新增建设用地指标与农业转移人口落户数量挂钩，加快提高户籍人口城镇化率，2020年全市城镇化水平达到55%。着力建设美丽乡村。积极开展“百村示范、千村创建、村村整治”行动，有计划、有步骤地推进美丽乡村建设。“十三五”时期，全市每年启动30个以上美丽乡村示范，到2020年，全市建成200个以上村容整洁、产业坚实、生态优美、乡风文明的美丽乡村，85%的行政村达到新农村建设要求。着力厚植生态优势。坚定不移推进“生态优先”，加强国土空间开发管控，推动形成绿色生产生活方式，强化生态环境治理与保护，强化节水节能减排等约束性指标管理，全面实行森林禁伐限伐，确保森林覆盖率稳定在65%以上，实施绿色GDP和生态环境损害考核，争创国家生态文明建设示范市，建设蓝天常有、青山常在、绿水长流的美丽永州。

　　打造改革开放新高地。继续走好“永州路径”。紧紧围绕增强发展活力、破解发展难题，加快实施重点领域和关键环节的改革，健全有利于创新发展的体制机制。着力深化行政管理体制改革、土地和农村产权制度改革、社会治理机制改革、结构性改革，始终以引领改革风气之先的智慧、胆识和气魄，进一步解放思想，先行先试，继续争当全省改革创新的先行区，赢取更多改革发展的新红利。坚定贯穿开放发展。全面对接和融入国家、省里区域发展战略，积极推进双向开放和内外联动，加大招商引资和跨区域合作，加强国际经贸合作，构建开放大平台和新机制，将永州打造成为湖南对接东盟桥头堡、“一带一部”重要战略支点和区域合作的新典范。大力推进民营兴市。进一步放宽投资领域、降低准入门槛、优化发展环境，努力扩大社会资本投入，着力激发市场主体活力，加快培育一批本土企业家群体和创新创业精英，构建“能人创企业、干部创事业、百姓创家业”的生动局面。引导支持非公企业转型创新发展，不断推动非公经济增长总量、提质量、强效益。

　　创造幸福美好新生活。实施脱贫攻坚工程。把脱贫攻坚作为头等大事，扎实抓好精准识贫、精准扶贫、精准脱贫各项工作，确保2020年现行标准下农村贫困人口全部脱贫，贫困县全部摘帽。促进教育优先发展。深入实施名师名校名校长工程，全面提高教育质量，着力推动义务教育均衡发展，全面普及高中阶段教育，逐步分类推进中等职业教育免除学杂费，积极发展学前教育，做大做强高等教育，扎实抓好成人教育、特殊教育、老年教育，支持发展民办教育。推进健康永州建设。深化医药卫生体制改革，强化基层医疗服务、疾控和卫生应急管理体系建设，做好疾病防控、妇幼保健、爱国卫生运动工作，巩固完善基本药物制度，逐步推行分级诊疗。积极发展中医药事业和医疗康体产业。大力支持社会力量办医。深入实施食品药品安全战略。积极创建“百岁健康市”。完善社会保障体系。实现城乡居民社会养老保险全覆盖。建立统一的城乡居民基本医疗保险制度，完善城乡居民大病保险制度。建立健全被征地农民社保制度。健全工伤保险、失业保险制度体系。逐步合并实施生育保险和基本医疗保险。完善社会救助服务，实现城乡社会救助全覆盖。推动就业创业发展。推进就业优先战略，完善就业创业服务体系，加强就业援助，开展贫困家庭子女、失学青少年、农民工、失地农民、失业人员、转岗职工、退役军人等免费接受职业培训行动，实施新生代农民工职业技能提升计划，完善创业扶持政策，鼓励以创业带动就业。推进人才强市战略。加快引进和培育一批高层次、复合型、创新性、国际化、急需紧缺型人才，着力发现、培养、集聚科技领军人才、企业家人才、高技能人才队伍，强化经济社会发展的人才、智力支撑。加强社会主义精神文明和民主法制建设，加强和创新社会治理，建设和谐、平安、幸福永州。

　　三、2016年工作安排

　　今年是“十三五”开局之年，也是我市深度实施“四个三”工作部署的关键之年。全市经济社会发展的主要预期目标是：地区生产总值增长9%，规模工业增加值增长10%，固定资产投资增长17%，社会消费品零售总额增长11%，财政总收入增长15%，城乡居民收入分别增长9%和10%以上，城镇登记失业率控制在4.5%以内，人口自然增长率控制在8.35‰以内，全面小康程度提升3个百分点，精准脱贫12万人以上，全面完成环境保护、资源节约、安全生产等约束性指标。

　　实现今年发展目标，我们要认真贯彻落实市委经济工作会议精神，紧紧围绕全面建成小康社会这个总目标，深入实施“四个三”工作部署，持续推进“八大计划”落实，加快释放改革红利，着力厚植生态优势，抓实抓牢“六件惠民实事”，全面提升民生福祉，努力为“十三五”开好局、起好步。

　　（一）突出扶贫攻坚，加速推进全面小康建设

　　大力实施精准扶贫。全面落实已出台的系列政策文件，用好社会保障托底政策。因地制宜推进重点产业扶贫项目，大力支持贫困村发展集体经济，开展好对贫困家庭劳动力实用技能、劳务就业等方面的培训，引导和支持有劳动能力的人通过就地创业就业实现脱贫。积极开展评级授信工作，加大金融扶贫的支持力度。把易地搬迁扶贫与生态环境保护结合起来，进一步加快搬迁项目进程。大力改善贫困地区办学条件，实施好“雨露计划”和“一家一”工程，对建档立卡贫困家庭中的“两后生”全面帮扶到位。加强贫困地区基层组织建设，组织好对驻村干部和贫困村主干的轮训，提高引领脱贫攻坚能力。

　　加快发展现代农业。突出农业标准化建设和示范，进一步健全农业产业标准体系、质量控制与监督制度，启动农产品质量安全追溯和信用体系建设，确保农产品农残监测合格率97%以上，新建农业标准化示范基地60个以上，新发展“三品一标”产品70个以上，争创国家地理标志保护产品示范区。加快培育现代农业园区，着力理顺体制机制，整合涉农资金，推动永州国家农科园做大做强，加强县区现代农业产业园区示范，推进祁阳国家现代农业示范园建设。优化提升主导产业，确保粮食播种面积825万亩，水果150万亩，蔬菜260万亩，烤烟28万亩，全面推进温氏集团在永项目建设，推动养殖产业转型升级。加快培育发展新型农业经营主体，推进供销标杆社、农民合作示范社建设。深入实施土壤肥力提升工程，实现测土配方施肥面积950万亩以上。加大对农村电商发展的政策扶持，引导鼓励电商、物流、商贸、金融、供销、邮政、快递等各类市场主体进驻农村，加快培育一批省、市级电子商务示范企业，新增100家电商企业落户，建设一批农村“星创天地”。推进落实阿里巴巴农村淘宝和“满天星·点亮中国”永州农产品质量防伪溯源体系项目。整合物流资源，大力发展第三方物流，构建方便快捷的农村物流体系。加强高端电商人才引进和本土电商人才培育。加强农村互联网技术应用和推广，推动农业企业加快“触电”。做好特色农产品包装和营销，打造一批知名农产品品牌。

　　培育壮大县域经济。认真落实促进县域经济发展的政策措施，切实加大对县域经济发展的支持力度，不断增强县域发展活力和整体实力。着力支持县区向上争取转移支付、申报项目资金、争创各类试点示范，引导鼓励市级金融机构到县区设立分支机构，加大对县区发展的信贷投入。推进扩权强县和扩权强镇，赋予县区和乡镇更多自主权。完善县域经济发展量化考核评价机制，积极鼓励创先争优。高度重视基层工作，继续抓好乡镇“五小”设施、社区办公用房、村级活动场所标准化建设，加大乡村社区运转经费保障力度，落实好县以下机关公务员职务与职级并行制度，保障好乡镇村组和街道社区干部的正常待遇。真情关心基层干部成长进步，逐步形成干部到基层锻炼、在基层成长、从基层选拔的培养机制，把更多优秀基层干部选拔到更加重要的岗位上来。进一步清理规范面向基层开展的各类检查、考核、评比、达标活动，切实为基层减负减压。

　　着力建设美丽乡村。突出规划引领，出台系列指导性文件，抓好“点、线、面”结合。照顾“面”，年内完成50%以上行政村村庄规划编制，因地制宜制定产业发展规划，引导农民主动参与美丽乡村建设。提高垃圾集中转运综合处理率，加大改水改厕改厨和污水处理力度，规范农村建房管理，加强农村危房改造和空心房清理整治工作，开展“点亮农村”行动。注重“线”，把铁路、国省道、高速公路沿线等视线范围内的村庄作为重点区域，率先予以突破。办好“点”，选择基础条件较好、交通优势明显、特色比较突出、群众积极性高的村庄（尤其是古村落）作为市县领导的联系点，示范带动全市美丽乡村建设。全年布点500个行政村的村庄整治，建设美丽乡村30个以上。

　　（二）突出改革引领，持续释放发展活力

　　准确把握中央全面深化改革的新要求新部署，全面完成省定改革任务，深入推进“3+2”重点改革。

　　深化行政管理体制改革。继续深化行政审批制度改革，对上级要求取消、下放的事项，一律取消下放，对已下放的事项必须落实到位。动态完善“三清单一目录”制度，全面推行政务服务事项“两集中两到位”改革和“一站式”服务，积极实施并联审批，变“多头受理”为“一口受理”。推进公共服务信息平台建设，促进办事部门相互衔接，变“群众来回跑”为“数据网上走、部门协同办”。理顺市县两级政府行政执法管理体制，推进执法重心和执法力量下移，加强食品药品、安全生产、环境保护、劳动保障等重点领域基层执法力量。扎实开展事业单位法人治理结构改革试点，推动条件成熟的生产经营类事业单位转为企业或社会组织。整合建立全市统一的公共资源交易平台。

　　深化农村产权制度改革。整合农口、规划建设、国土等力量，完成乡镇一级农地利用规划编制工作。规范农村产权交易平台，建立完善县、乡、村三级网络体系和科学规范的产权交易规则，全面开展农村产权交易业务。加快农村土地流转，提升规模化和机械化生产水平。大力推动农村土地承包经营权抵押贷款，大胆探索农建投等农村投融资新途径，开展以村集体经济股份制为主要内容的农村集体经济组织产权制度和农村宅基地入市改革试点，为发展现代农业注入新的活力。

　　深化社会治理机制改革。不断完善“条块结合、以块为主”的网格化管理体制机制，强化社区工作保障，健全社区工作运行机制，进一步下放服务事项，将社区做强，把工作干实，畅通服务群众“最后一公里”。充分发挥自治组织和各类社会组织在基层社会治理中的作用，形成党政善治、社会共治的新格局。扎实抓好合村改革，确保上半年完成目标任务。结合农村改革工作，出台农村网格化建设标准和工作流程，加快建设步伐，让城乡居民共享便捷服务。

　　积极推进结构性改革。围绕中央提出的去产能、去库存、去杠杆、降成本、补短板五大任务，在供需两侧共同发力，不断增强经济发展原动力。扎实推进供给侧改革。坚持按经济规律办事，减少政府对微观经济活动的不当干预，减轻企业负担，发挥市场在资源配置中的决定性作用，让各类要素自由流动，不断创造新的经济增长点。深入推进国有企业改革，打好国企改革扫尾攻坚战，全面完成第二轮国企改革扫尾任务，清理处置“僵尸企业”，积极化解过剩产能，腾出宝贵的实物资源、信贷资源和市场空间，支持优势企业转型升级；完善国有资产监管体系，加快建立现代企业制度。加强财税和投融资体制改革，积极支持税收征管体制改革，推进“营改增”，全面落实结构性减税，引导企业转型和产业集中发展；做好政府存量债务置换，抓好呆账资金清理；加强财源建设和财税征管，提升财税收入质量和生财聚财理财水平；积极扩大有效信贷投入，全年新增银行贷款165亿元以上，市级两大融资平台融资100亿元以上；完善地方金融体系，确保永州农商行上半年挂牌营业，新引进1家股份制银行和1家村镇银行。扩大直接融资，力争2家企业在新三板挂牌；发挥财政资金的放大效应，更大规模带动和激活民间投资，巩固扩大PPP项目试点成果；创新规范各类担保公司运行，严厉打击非法集资活动，有效防范金融风险。有序推进需求侧改革。充分发挥投资的关键作用，在补齐软硬短板上挖掘投资需求、降低投资门槛、提高投资效益；充分发挥消费的基础作用，积极培育教育文化、健康养老、体育健身、旅游休闲、信息、汽车、绿色等消费热点。多措并举搞活住房消费，合理消化房地产库存，推动房地产市场健康稳定发展。

　　（三）突出转型升级，培育壮大新的经济增长点

　　深入推进园区建设。以创建“百亿园区”为目标，确保园区规模工业总产值增长20%以上，园区主导产业产值占比达到40%以上，实现宁远、江华工业园规模工业总产值过100亿。突出标准厂房建设。全力实施创新创业“135”工程，全年新增标准厂房面积150万平方米，引进“双创”企业190家。着力建设专业园区。大力培育特色产业集群，积极引导电子信息、生物医药等产业向园区集中，在长丰工业园建设汽车配套产业专业园区，支持县区一批专业特色产业园做大做强。推进产城融合。进一步理顺园区体制机制，实施扩权强园，完善园区规划和产业布局，避免同质化竞争。开展“园区基础设施建设年”活动，重点加快园区道路、水电管网建设和成片征地拆迁力度，不断完善园区生活设施配套，促进城市公共服务向园区延伸。

　　深入推进规模工业企业转型升级。大力培育新兴产业。重点发展先进装备制造、电子信息、生物医药、新能源新材料等战略性新兴产业。着力以现有产业、企业为基础，加大龙头企业和配套企业引进力度，不断拉长产业链条，打造产业集群。扶持壮大骨干企业。加大对现有优势企业的扶持力度，盘活存量，做大增量。支持长丰汽车15万辆整车生产线建设，培植零陵烟厂、康都制药、奔腾彩印、果秀食品、福嘉有色、九恒集团、坤昊实业、凯盛鞋业等骨干企业做大做强。全年新增规模企业100家以上，新增主营业务收入过亿元企业20家、5亿元以上企业5家、10亿元和50亿元以上的企业各1家。启动实施一批重大技改升级项目。积极对接“中国制造2025”，制定实施永州先进装备制造五年行动计划，对农产品、矿产品等精深加工产业进行改造提升，全年完成工业技改投资250亿元。积极实施“互联网+”改造传统产业，支持企业通过挂牌上市、兼并重组、技术改造等路径，整合市场优势资源，加快实现转型升级。

　　深入推进创新创业。把创新创业作为引领后发赶超的新动力，推动形成大众创业、万众创新的新态势。全年新增市场主体1.5万个，新设立企业3000家，新培育小微企业1300家以上。大力“清障”。继续深化商事制度改革，抓好“先照后证”后置行政审批、后续市场监管和企业信用体系建设，加快构建众创、众包、众扶、众筹“四众”支撑平台，通过舆论引导、典型示范、政策扶持，营造良好的“双创”氛围。全面“减负”。开展降低实体经济企业成本行动，进一步整顿规范中介服务组织，推进行业协会商会与行政机关脱钩，清理各种不合理收费，精简归并“五险一金”，着力降低企业制度性交易成本、税费负担、社会保险费、财务成本、电力价格以及物流成本，切实为企业减负减压。积极“搭台”。加强创业型城市建设，抓好“双百工程”，深入开展创新创业带动就业示范县区和省级创新创业示范基地建设，每个县区和永州经开区、永州国家农科园建设一个创新创业孵化园。全年新增城镇就业5.5万人、农村劳动力转移就业4.7万人。科技驱动。实施省级以上科技项目30个以上，新认定高新技术企业5家以上，专利授权800件以上，全社会研发经费入统增长35%，新增1家省级以上企业工程技术研究中心。

　　深入推进文化旅游产业发展。推进公共文化服务标准化、均等化，完善文化基础设施网络，重点抓好市文化艺术中心、市民中心等项目建设。加快九嶷山5A、舜皇山4A景区申创步伐，确保永州植物园、周家大院、江华香草源等成功创建3A景区。加大非物质文化遗产保护力度，传承永州历史文脉。加快推进“零陵古城”文化旅游综合保护开发项目，进一步推进湖湘风情文化旅游小镇和其它特色乡镇创建，结合美丽乡村建设和精准扶贫工作，重点扶持发展一批乡村旅游示范村，推动大众休闲旅游、体验式旅游蓬勃发展。

　　深入推进开放型经济发展。积极扩大招商引资。突出重点区域、重点产业、重大项目招商，继续抓好小分队招商，积极鼓励以商招商、老乡招商和永州异地商会招商，办好首届永商大会。健全项目会审机制，强化签约项目跟踪落地，全年实际利用外资8.5亿美元，内联引资215亿元。加快推进外贸发展。充分用好示范区、桥头堡优势，加大企业走出去力度。大力支持发展跨境电子商务，让外贸企业更好实现“全球买、全球卖”。继续实施外贸企业“破零倍增”计划，重点扶持“五十百”企业做大做强，全年实现外贸进出口9亿美元。持续优化通关平台。加大永州内陆港及配套物流园区推进力度，做好湖南农副产品集中验放场二期前期工作，完成湘南进出口食品、农副产品检测中心建设。促进永州电子口岸与关检“三个一”、单一窗口融合发展，加快国家一类公路口岸申报。开通到香港口岸直通车。

　　（四）突出交通先行，继续突破基础设施瓶颈

　　加强交通建设。扎实推进三年交通大建设行动，建成国道322祁阳、零陵绕城线及国道207道县绕城线、省道236宁远至中和、省道239江永潇浦至广西灌阳边界、省道238东安芦洪市至小江口等公路，加快省道355江华分水岭至白芒营、省道348祁阳黎家坪至冷水滩马坪、省道228新田竹林坪至县城等公路建设进度，开工建设省道356江华沱江至涔天河一级公路、宁远柏家坪至道县柑子园、道县上关至湘源温泉等项目，完成农村公路改造1000公里，建成农村招呼站200个，探索建立农村公路养护长效机制。加速推进零陵机场迁建、衡永高速、邵永客专、永州铁路枢纽等项目前期进度。

　　加强水利建设。抓好涔天河水库扩建工程、灌区工程、毛俊水库和东安芦江水库、宁远金钩挂水库、江华清江水库建设。完成552座小型水库、3座中型水库、2座中型水闸除险加固任务，扎实推进小农水重点县、中小河流治理、水土保持、灌区续建配套等水利项目建设。新增农村饮水安全人口30万以上。

　　加强能源建设。积极推进神华国华永州电厂一期工程建设，做好二期前期工作。加大电能替代、清洁替代力度，加快风电开发，今年建设30万千瓦、开展前期工作30万千瓦。推进中心城区垃圾焚烧发电、东安、新田及华威福田光伏发电等项目建设。启动城市配电自动化建设，推进中心城区110千伏网络优化工程，抓好农村电网改造升级。积极对接省天然气长输管线，加快“气化永州”进程。

　　加强智慧永州建设。加强信息基础设施建设。统筹规划城乡通信基础设施，推进光纤入户和农村行政村宽带全覆盖，重要场所实现wifi全覆盖。完善基础数据库。积极构建永州数据云，进一步完善基础数据库，推动政府部门数据共享和数据集成运用。加快北斗导航（湖南）数据中心建设。加快平台应用。充分利用智慧永州综合运用平台，对接开发智慧交通、智慧旅游、智慧医疗、智慧教育、智慧市场监管等应用系统，抓好公共服务、社会治理、数字城管等子系统的深度开发。谋划数据产业发展。制定大数据产业发展规划，深入实施“互联网+”行动计划、“数字企业”示范工程和企业信息化“登高计划”，大力推动大数据与云计算、物联网、移动互联网等新一代信息技术融合发展，促进传统产业数字化、网络化、智能化。

　　（五）突出核心带动，加快构建新型城镇发展格局

　　尊重城市发展规律，坚持以人民为中心的城市发展思想，加快推进中心城市扩容提质三年行动，带动县城和小城镇竞相发展，形成具有永州地域特色的城市发展格局。

　　加快中心城区连城扩容和配套提质。加快“一江两岸、东扩西跨、南北联城、中部崛起”建设步伐。完善城市基础设施和功能配套，启动重点建设项目140个，完成投资96亿元。加强地下管网改造、永州大道提质改造、湘江西岸棚户区和城中村改造、河东河西城市综合体、万商红商贸城等项目建设，加大河西路网、给排水管网、垃圾收运等设施的建设改造力度。大力开展控违拆违行动，加强沿江风光带、沿路景观带和城市小游园建设。积极推进城市公交优先战略，加大快速公交建设和绿色公交推广力度，抓好公交体制改革。不断完善城市管理和服务，巩固提升国家卫生城市、国家森林城市创建成果，继续做好创名城、创交模工作，启动国家园林城市、全国文明城市创建工作。着力谋划重大节庆、会展等活动，争取全国性训练基地或赛事落户永州，打造新的城市名片。

　　着手打造潇湘城市群。坚持规划引领、交通先行，加快完成城市群总体规划及交通、产业、生态等专项规划编制，制定城市群建设的时间表、任务书和路线图。积极推进冷东快速通道、零双一级公路建设，新建二广高速上岭桥互通至泉南高速大忠桥互通连接线，打造潇湘城市群半小时交通圈。

　　提升县城和小城镇综合发展水平。以并乡合村为契机，加强县城和乡镇建设，推进土地利用规划修编，加强城镇路网、供水供气管网、垃圾污水处理设施、公交站点、公共停车场、地下综合管廊等市政基础设施和公共服务设施建设，推进有条件的县城“改镇设办”。支持各县争创国家、省级园林城市、卫生城市、文明城市，支持更多县、镇进入国家、省级新型城镇化试点和城乡一体化示范。加强中心镇扩权强镇改革，探索小城镇建设多渠道融资途径，抓好18个国家、省级重点乡镇示范。

　　（六）突出实事惠民，全面提升民生福祉

　　落实社会政策要兜底的硬性要求，着力增强人民群众的获得感、满意度和幸福指数。

　　实施生态惠民。完善和落实主体功能区规划，实施生态功能区产业准入负面清单，坚决杜绝“两高一资”项目。加强林业资源管护和野生动植物、自然保护区及湿地保护，新创一批森林公园、湿地公园，加快森林植物园建设。深入推进“水气同治”，加强饮用水水源地保护，完成县级以上饮用水源保护区陆域范围划定。实施湘江保护和治理第二个三年行动计划，深化湘江流域污染防治，全面整治宋家洲及周边生态环境。积极争取国家、省土壤重金属污染修复示范项目。开展重点区域、重点行业、重点企业环境污染整治和有色、化工等行业强制性清洁生产审核，建设高污染燃料禁燃区，依法淘汰和关闭高污染、高能耗生产工艺。加快淘汰落后养殖产能，全面禁止小二型以上水库投肥养鱼和河流电鱼、炸鱼、毒鱼。推进城市环境综合治理，加快生活污水处理设施建设和改造升级，推动工业园区污水集中处理设施配套建设。落实环保执法垂直管理制度改革，加大依法打击环境违法行为力度。大力发展低碳、循环经济，积极策划包装优良生态资源，推动建立以湘江源头为重点的生态补偿机制。培育、发展生态交易市场，争取在永州试点建立湖南地区碳汇交易、节能量交易、排污权交易、水权交易平台。深入推进造林绿化，抓好矿山植被恢复，完成人工造林35万亩。

　　推进教育强市。进一步落实学校和校长办学自主权，加快实现2-3所示范性高中进入全省名校第一方阵，在每个学段打造一批品牌学校。鼓励龙头学校托管中学、小学，放大优势教育资源。积极改善办学条件，年内新建义务教育合格学校60所、标准化学校30所、农村公办幼儿园20所、农村教师公租房2000套，新增中心城区义务教育学位8000个，努力化解“大班额”。加强学校食堂及周边环境整治，打造良好教书育人环境。

　　完善社会保障。全面推进“五险”改革统一征缴进程，建立基本养老金合理调整机制，推进机关事业单位养老保险制度改革，落实园区企业打捆实施养老保险费率过渡试点政策。加快推进医保城乡统筹，深化医保付费制度改革，推动县级公立医院改革向纵深发展。稳步提高城乡低保、五保供养、优抚对象等群体的救助水平，做好孤儿、重残智残、失独家庭等群体的救助工作，健全完善临时救助制度。加快社会化养老服务体系建设，办好一批示范养老机构。完善残疾人保障服务体系，落实好扶残助残的各项政策。保障妇女、儿童权益。切实关爱留守儿童、孤寡老人、流浪乞讨人员等弱势群体。重视和发展慈善事业。做好水库移民搬迁安置和后期扶持工作。

　　发展各项事业。全面实施公民道德建设工程，深化各类群众性精神文明创建活动。净化未成年人健康成长社会文化环境。启动“七五”普法，增强全社会尊法学法守法用法观念。深化政务公开和办事公开，加强政府网站建设和管理，提升服务办事能力。推进文化惠民工程，加强文化市场监管和广电新闻出版管理。启动《永州市志》编修工作。开展全民健身运动，提高竞技体育水平。加强卫计工作，落实“全面两孩”政策，加大人口出生缺陷综合防控，抓好湘雅永州医院建设。加强知识产权保护和运用，推广普及科技知识。加强防灾减灾体系建设，提升防灾抗灾能力。发展老龄事业，做好老干部工作。落实民族、宗教、侨务政策，促进民族地区加快发展和宗教领域和谐稳定。

　　抓牢六件惠民实事。棚户区改造。支持列入省保障性安居工程示范项目的湘江西岸棚户区改造和祁阳嘉园公租房建设，推动政府购买棚改服务和货币化安置，全年完成保障性安居工程建设4.19万户，其中城市棚户区改造3.15万户。完成农村危房改造1万户。小区管理。完成中心城区小区整治突出问题的扫尾工作，加快建章立制步伐，不断巩固提升整治成果。规范小区管理，各县区按时完成三年整治工作任务。农村环境治理。积极落实农村环境综合整治整县推进项目，加强农村面源污染治理、污水治理、垃圾集中处理和村容村貌整治工作，农村生活垃圾集中处理率达到90%，卫生厕所普及率达到72.5%，18%的行政村实现生活污水处理，湘江流域范围内纳入整治计划的117家养殖场粪污治理100%达标。放心食用油和米粉。继续抓好食用油、米粉放心工程，完善放心米粉示范店（企业）标准。严厉打击非法生产加工销售地沟油、潲水油及其它劣质食用油、米粉黑窝点，加快推进食用油、米粉生产加工企业和小作坊提质改造。五小水利建设。全年投资4亿元以上，实施“五小水利”工程7310处，解决750个行政村农田灌溉“最后一公里”问题。社会安全。坚持打击开路，防范为本，创新和完善立体式治安防控体系，全面加强反恐工作，切实维护网络社会安全，严厉打击严重暴力犯罪、盗骗毒犯罪、“三强三堵”犯罪和农村偷盗行为，深入开展治安乱源村、治安失控村摸排整治，从严查处信访活动中的违法犯罪行为，全力维护政治稳定和社会治安大局稳定，不断提升人民群众安全感和满意度。创新矛盾纠纷化解方式，完善人民调解、行政调解、司法调解联动工作体系，认真落实领导干部接访、下访和包案处理信访工作制度，推进依法信访、网上信访。积极开展“打非治违”，全面整改各类安全隐患，坚决杜绝重特大安全事故发生。

　　推进军民融合。坚决支持深化国防和军队改革，主动帮助解决好退役军人、职工安置工作，推动经济建设和国防建设融合发展，充分发挥国防后备力量保一方平安促一方发展的作用。

　　各位代表，踏上新征程，肩负新使命，我们将严格按照“四个全面”和“三严三实”的要求，更加自觉抓好政府自身建设，更好担当人民赋予的神圣职责。

　　牢记为政必为。强化责任意识，始终把发展作为第一要务，把事业作为第一追求，把老百姓的事作为天大的事，一心一意谋发展，聚精会神搞建设。增强担当精神，敢于在加快发展中担重任，在深化改革中涉险滩，在扶贫攻坚中啃硬骨头，对市委作出的决策部署，雷厉风行、坚决执行；对市政府抓的事情，议立决、决立行，不争论、不折腾，定一件办一件，办一件成一件。狠治懒政庸政，深入推进机关效能建设，加大督查问责力度，开展“为官不为”专项整治，着力解决慢作为、不作为、乱作为等问题，营造肯干事、能干事、干成事的浓厚氛围。

　　学会为政善为。深入解放思想，坚决破除思维定势、工作惯性、路径依赖，面对新常态，面对新矛盾、新问题，善于在变中求新、变中求进、变中突破。加强学习锤炼，自觉把学习作为一种习惯、一种追求，不断优化各级干部的知识结构和专业结构，提高引领发展的科学化、法治化、专业化水平。创新思维理念，牢固树立“五大发展”新理念，切实强化人本思维、法治思维、超前思维、高铁思维、信心思维、融合思维、“互联网＋”思维、起跑线思维，提高运用创新思维深化改革、推动发展、化解矛盾、维护稳定的能力。

　　推进为政实为。坚持求真务实，深入开展调查研究，严格落实各级领导联系重点项目、重点企业、重大工作制度，坚决摈弃以会议落实会议、以文件贯彻文件、以形式主义反对形式主义的官场陋习。严格科学决策，完善公众参与、专家论证、风险评估、合法性审查、集体讨论决定相结合的决策体系，落实重大决策终身责任追究制度及责任倒查机制，增强决策的可行性和有效性。强化考核奖惩，抓好干部队伍执行力考核、绩效考核制度的完善落实，强化考核结果运用，充分发挥考核导向作用，切实把各级干部的思想和精力集中到抓工作、干事业、谋发展上来，进一步形成你追我赶、创先争优的浓厚氛围。

　　做到为政廉为。推进依法行政，带头维护宪法和法律权威，严守政治纪律和政治规矩，自觉在市委的绝对领导下开展工作，严格规范公正文明执法。自觉执行人大及其常委会的决议决定，支持和保障人民政协履行职能。认真办理人大代表建议和政协委员提案，虚心听取和采纳各民主党派、工商联、无党派人士、人民团体的意见建议。完善政府内部层级监督和专门监督，继续强化行政监察和审计监督，积极推行政府法律顾问制度。加强正风肃纪，坚决执行《中国共产党廉洁自律准则》、《中国共产党纪律处分条例》，严格落实中央八项规定、省委九项规定和市委十项规定，从严从紧控制“三公”经费，全面完成公车改革。狠抓反腐倡廉，落实“一岗双责”，坚决查处官商勾结、权钱交易、权色交易、权权交易等腐败问题，加大苍蝇式腐败治理力度，切实做到干部清正、政府清廉、政治清明。

　　各位代表，宏伟蓝图已经绘就，壮丽前景催人奋进。让我们紧密团结在以习近平同志为总书记的党中央周围，在市委的坚强领导下，万众一心，开拓奋进，为夺取全面建成小康社会决胜阶段的伟大胜利、创造630万永州人民更加幸福美好的新生活而努力奋斗!
